# Brain atrophy in NMOSD and MOGAD: a meta-analysis of volumetric and DTI biomarkers

**DOI:** 10.3389/fneur.2025.1703283

**Published:** 2025-12-02

**Authors:** Ariel Rechtman, Omri Zveik, Adi Vaknin-Dembinsky

**Affiliations:** 1 Department of Neurology and Laboratory of Neuroimmunology and the Agnes-Ginges Center for Neurogenetics, Hadassah-Hebrew University Medical Center, Jerusalem, Israel; 2 Faculty of Medicine, Hebrew University of Jerusalem, Jerusalem, Israel

**Keywords:** NMOSD, MOGAD, volumetry, meta-analysis, atrophy

## Abstract

**Background:**

Neuromyelitis optica spectrum disorder (NMOSD) and myelin oligodendrocyte glycoprotein antibody-associated disease (MOGAD) are demyelinating diseases of the central nervous system. Brain atrophy is well recognized in multiple sclerosis; however, approximately 50% of studies report no significant difference in overall brain volumes when comparing NMOSD patients with healthy controls (HCs). To quantitatively assess differences in brain volume and white matter integrity in NMOSD and MOGAD patients compared to HCs through a meta-analysis.

**Methods:**

A systematic literature search of English articles in PubMed was performed through December 2024. We analyzed sixty-one studies that met the inclusion criteria, providing volumetric MRI or diffusion tensor imaging data with HC comparisons. Outcomes of interest included brain volume, and DTI parameters. Standardized mean differences were computed, and random-effects meta-analyses were performed to account for study heterogeneity.

**Results:**

The studies included data from 1,786 NMOSD patients, 376 MOGAD patients, and 1,936 HCs. NMOSD patients exhibited significantly lower total brain, gray, and white matter volumes compared to HCs. Notable atrophy was observed in several regions including the accumbens, brainstem, caudate, cerebellum, hippocampus, putamen, and thalamus. MOGAD patients have reduced brain volume compared to HCs. Furthermore, comparisons demonstrated that NMOSD patients had significantly lower brain and gray matter volumes than MOGAD patients.

**Conclusion:**

Our meta-analysis confirms substantial brain atrophy in NMOSD patients compared to both HCs and individuals with MOGAD, indicating a more pronounced neurodegenerative impact than previously recognized. These findings carry important clinical implications by enhancing our understanding of disease-specific imaging biomarkers.

## 1 Introduction

Neuromyelitis optica spectrum disorder (NMOSD) is a chronic inflammatory autoimmune astrocytopathy disease of the central nervous system (CNS) characterized by optic neuritis (ON) and transverse myelitis (TM) (1, 2). NMOSD is most commonly associated with a pathogenic serum IgG antibody targeting the water channel aquaporin-4 (AQP4) (3). Although NMOSD is not a primary demyelinating disorder, astrocyte damage is followed by secondary demyelination and neuronal injury (4, 5). There are patients that have typical NMOSD symptoms in the absence of this antibody and are classified as seronegative NMOSD (6). Notably, a subset of these patients has since been reclassified as having myelin oligodendrocyte glycoprotein antibody-associated disease (MOGAD), following the identification of antibodies against myelin oligodendrocyte glycoprotein (MOG), a protein located in the myelin sheath (7, 8).

Magnetic resonance imaging (MRI) has revolutionized the diagnostic approach to demyelinating disorders (9, 10). It enables visualization of both active and inactive lesions across the CNS and plays a role in assessing disease progression and therapeutic response (11). Volumetric MRI, which assesses changes in brain and spinal cord volumes, is particularly valuable (12, 13). It provides quantitative data that reflect the extent of neurodegeneration, which is crucial for predicting long-term outcomes such as disability (14).

NMOSD exhibits a diverse range of brain MRI findings that differ markedly from those observed in multiple sclerosis (MS) and MOGAD (15). Notably, up to 40% of patients exhibit normal or only non-specific brain imaging at onset (16). When present, lesions often include unique “ground glass”-like heterogeneous lesions in the corpus callosum, which may help differentiate NMOSD from MS (17). Hypothalamic involvement, characterized by high T2/FLAIR signal intensities extending into the adjacent parenchyma and third ventricular walls, is another distinguishing feature. Although gadolinium-enhancing lesions are rare in NMOSD, a characteristic “cloud-like” enhancement patternis occasionally observed (18). Cerebral T2 lesions in NMOSD tend to shrink over time, often leaving residual high signal intensity, unlike MS, where lesions commonly persist, or MOGAD, where they frequently resolve (15). Importantly, lesions in the posterior medulla corresponding to area postrema syndrome are considered highly specific for NMOSD, as are abnormalities in the peri-aqueductal gray, nucleus tractus solitarius, and floor of the fourth ventricle (19). Brainstem and cerebellar lesions are less common overall, though dentate nucleus involvement may occur (17).

Diffusion tensor imaging (DTI) is an advanced MRI technique that tracks the diffusion of water molecules in tissue, revealing the microstructural integrity of white matter tracts (20). In demyelinating disorders, DTI provides invaluable insights into the extent of white matter damage beyond what is visible on standard MRI scans (21). It quantifies damage in terms of reduced diffusion anisotropy, which can be indicative of axonal loss and demyelination (20).

In NMOSD, DTI studies have demonstrated significant white matter abnormalities even in regions appearing normal on conventional MRI scans (22). These abnormalities include reduced fractional anisotropy and increased mean diffusivity, suggesting widespread disruption of normal white matter integrity (23).

Investigations into brain volume differences between NMOSD patients and healthy controls (HCs) have yielded inconsistent results. While approximately half of the studies report significant brain atrophy in NMOSD, others find no measurable differences compared to HCs. This meta-analysis aims to reconcile these divergent findings by conducting a comprehensive review and synthesis of the available literature on brain volume in NMOSD and MOGAD patients.

## 2 Methods

### 2.1 Search strategy

A systematic review of peer-reviewed, English-language articles from the PubMed database was conducted, utilizing the search terms “NMOSD,” “NMO,” “AQP4,” “MOG-IgG,” “MOGAD,” “MOG” without limiting the year of publication. The most recent literature search was completed in December 2024. Initial screening based on titles and abstracts led to the selection of articles pertinent to this study, followed by an in-depth analysis of the full texts. The reference lists of the selected articles were also screened for additional relevant studies; however, gray literature was not included.

### 2.2 Selection criteria

Information was extracted on DTI parameters, fractional anisotropy (FA), mean diffusivity (MD), axial diffusivity (AD), and radial diffusivity (RD) within normal-appearing white matter (NAWM), as well as total brain volume, gray matter volume, white matter volume, and volumes of various brain tissues including: accumbens, amygdala brainstem, caudate, cerebellum, hippocampus, pallidum, putamen and thalamus.

Inclusion criteria comprised articles presenting data on brain volumes or DTI metrics in NMOSD and MOGAD patients. Articles were selected for inclusion in one or more of three pairwise comparisons: MOGAD vs. NMOSD, MOGAD vs. HCs, and NMOSD vs. HCs. Studies reporting data on all three groups were included in all comparisons, whereas studies presenting data for only two groups were incorporated solely into the relevant pairwise comparison. Exclusion criteria were applied to non-English articles, or studies with incomplete data. The NMOSD patient group included individuals diagnosed with NMOSD who were either positive or negative for AQP4-IgG antibodies.

### 2.3 Data extraction

Two researchers independently screened the resulting articles initially based on title, abstract and then full text. Articles were excluded based on predefined eligibility criteria with discrepancies resolved by consensus. Two researchers independently extracted data from the included studies using a standard form. The type of data that were extracted included volumetric information, DTI information and the number of participants. Where data were not available, the authors of these studies were contacted to request the data.

### 2.4 Data analysis

For each pairwise comparison, the standardized mean difference (SMD) was calculated to quantify the effect size. The pooled standard deviation was computed from the standard deviations of the two groups. The corresponding variance for each SMD was estimated to account for sampling error. Random-effects meta-analyses were performed using the restricted maximum likelihood (REML) method implemented in the metafor package. This approach was chosen to account for potential heterogeneity across studies. Heterogeneity was assessed with the *I*^2^ statistic, and studies were combined for three comparisons: NMOSD vs. HC, MOGAD vs. HC, and MOGAD vs. NMOSD. Sensitivity analysis, through the leave-one-out approach, was utilized to determine the influence of individual studies on the overall meta-analytic effect. The risk of publication bias was assessed by visually inspecting funnel plots and Egger’s test (24). A *p*-value of less than 0.05 was determined as statistically significant.

## 3 Results

### 3.1 Search results

**Figure 1:**
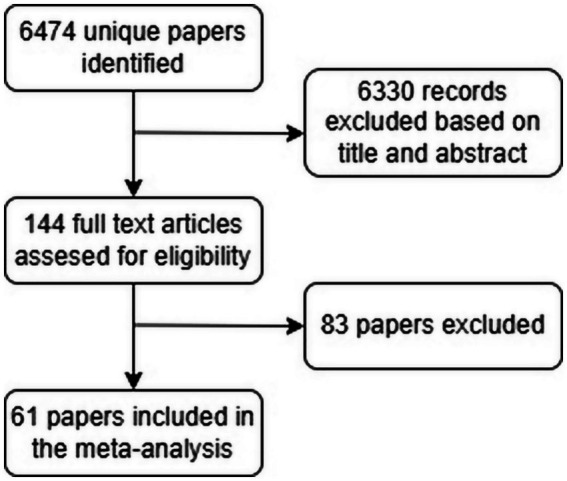
Flowchart of the study.

Our systematic search identified 6,469 records across pubmed and an additional 5 studies through manual searches. The entries were screened by title and abstract, leading to the full-text assessment of 144 articles. Of these, 83 were excluded: 55 did not report any brain volume data, 23 mentioned brain volume without providing numerical values, and 5 included data for only one group. Ultimately, 61 articles met the eligibility criteria and were included in this meta-analysis (Figure 1). In those articles were a total of 1786 NMOSD, 376 MOGAD patients and 1936 HCs (Table 1). Regarding age and sex differences between groups, most included studies either matched patients and controls on these variables, normalized results for head size, or reported no significant differences in age or sex.

**Table 1:** All studies included in this meta-analysis.

First Author	Publication year	Participants	Mean age	Female (%)	HC vs. NMOSD volumetric	HC vs. MOGAD volumetric	MOGAD vs. NMOSD volumetric	HC vs. NMOSD DTI
Rocca MA (37)	2004	HC: 15 NMOSD:10	HC: 48.30 NMOSD: 48.1	HC: 60% NMOSD: 60%	Yes			Yes
Pichiecchio (38)	2011	HC: 17 NMOSD:8	HC: No Information NMOSD: 50	HC: No Information NMOSD: 100%				Yes
Liu Y (39)	2011	HC: 27 NMOSD:27	HC: 34.20 NMOSD: 35.10	HC: 93% NMOSD: 93%				Yes
Blanc F (40)	2012	HC: 28 NMOSD:28	HC: 42.00 NMOSD: 45.30	HC: 68% NMOSD: 68%	Yes			
Chanson JB (41)	2013	HC: 30 NMOSD:30	HC: 41.70 NMOSD: 46.20	HC: 63% NMOSD: 63%	Yes			
Calabrese M (42)	2012	HC: 30 NMOSD:30	HC: 42.30 NMOSD: 42.60	HC: 70% NMOSD: 70%	Yes			
Sánchez-Catasús CA (27)	2013	HC: 15 NMOSD:15	HC: 46.60 NMOSD: 41.00	HC: 87% NMOSD: 87%	Yes			
von Glehn F (43)	2014	HC: 34 NMOSD:21	HC: 42.00 NMOSD: 38.00	HC: 82% NMOSD: 90%	Yes			
Weier K (44)	2015	HC: 34 NMOSD:30	HC: 31.40 NMOSD: 33.50	HC: 88% NMOSD:87%	Yes			
Liu Y (45)	2015	HC: 35 NMOSD:35	HC: 32.23 NMOSD: 35.40	HC: 80% NMOSD:89%	Yes			
Zhang LJ (46)	2015	HC: 26 NMOSD:49	HC: 45.30 NMOSD: 47.60	HC: 86% NMOSD:88%	Yes			
Liu Y (47)	2015	HC: 40 NMOSD:39	HC: 33.70 NMOSD: 36.90	HC: 70% NMOSD:89%	Yes			
Liu Y (48)	2015	HC: 27 NMOSD:54	HC: 51.22 NMOSD: 49.39	HC: 81% NMOSD:89%	Yes			Yes
Pache F (49)	2016	HC: 21 NMOSD:21	HC: 44.80 NMOSD: 44.70	HC: 86% NMOSD:86%	Yes			
Schneider R (50)	2017	HC: 26 NMOSD:18	HC: 39.7 NMOSD: 42.1	HC: 46% NMOSD:78%	Yes			
Finke C (51)	2016	HC: 36 NMOSD:36	HC: 45.4 NMOSD: 47.9	HC: 94% NMOSD:94%	Yes			
Streitberger KJ (52)	2017	HC: 17 NMOSD:15	HC: 46.00 NMOSD: 48.00	HC: 82% NMOSD:80%	Yes			
Qian W (53)	2016	HC: 18 NMOSD:13	HC: 43.40 NMOSD: 44.20	HC: 61% NMOSD:87%	Yes			
Hyun JW (54)	2017	HC: 44 NMOSD:91	HC: 39.00 NMOSD: 36.00	HC: 89% NMOSD:90%	Yes			
Su L (55)	2016	HC: 18 NMOSD:26	HC: 48.11 NMOSD: 47.00	HC: 83% NMOSD:81%	Yes			
Kim SH (56)	2017	HC: 43 NMOSD:93	HC: 39.00 NMOSD: 37.00	HC: 88% NMOSD:83%	Yes			
Liu Y (57)	2018	HC: 24 NMOSD:25	HC: 34.60 NMOSD: 35.60	HC:75% NMOSD: 84%	Yes			
Tian DC (58)	2018	HC: 24 NMOSD:36	HC: 40.50 NMOSD: 41.40	HC:92% NMOSD: 83%	Yes			
Kim SH (59)	2017	HC: 44 NMOSD:73	HC: 38.60 NMOSD: 36.80	HC: 89% NMOSD:96%	Yes			Yes
Sun J (60)	2017	HC: 30 NMOSD:36	HC: 47.40 NMOSD: 47.23	HC: 73% NMOSD:81%	Yes			Yes
Lee CY (61)	2018	HC: 18 NMOSD:13	HC: 43.40 NMOSD: 44.20	HC: 50% NMOSD:85%	Yes			
Rueda-Lopes FC (62)	2018	HC: 19 NMOSD:28	HC: 44.10 NMOSD: 38.50	HC: 74% NMOSD:72%	Yes			
Rocca MA (63)	2019	HC: 30 NMOSD:28	HC: 42.30 NMOSD: 42.40	HC: 70% NMOSD:79%	Yes			
Kuchling J (64)	2018	HC:26 NMOSD:23	HC: 43.70 NMOSD: 46.70	HC: 85% NMOSD:87%				Yes
Chen X (65)	2019	HC:26 NMOSD:30	HC: 36.69 NMOSD: 41.70	HC: 92% NMOSD:96%	Yes			
Savoldi F (66)	2020	HC:30 NMOSD:25	HC: 42.30 NMOSD: 43.40	HC: 70% NMOSD:76%	Yes			
Papadopoulou A	2019	HC:37 NMOSD:39	HC: 47.80 NMOSD: 50.10	HC: 87% NMOSD:92%	Yes			
Pudlac A (67)	2020	HC:20 NMOSD:20	HC: 50.00 NMOSD: 48.00	HC: 80% NMOSD:80%	Yes			
Tian DC (68)	2020	HC:55 NMOSD:90	HC: 46.10 NMOSD: 45.30	HC: 78% NMOSD:84%	Yes			
Asseyer S (69)	2020	HC:37 NMOSD:32	HC: 47.80 NMOSD: 51.10	HC: 87% NMOSD:97%	Yes			
Heine J (70)	2020	HC:30 NMOSD:30	HC: 44.00 NMOSD: 45.50	HC: 90% NMOSD:90%	Yes			
Duan Y (71)	2021	HC:60 NMOSD:38 MOGAD:35	HC: 36.90 NMOSD: 37.70 MOGAD:36.40	HC: 52% NMOSD:84% MOGAD: 60%	Yes	Yes	Yes	Yes
Messina S (72)	2022	HC:18 NMOSD:19 MOGAD:20	HC: 38.90 NMOSD: 55.60 MOGAD:41.80	HC: 56% NMOSD:68% MOGAD: 50%	Yes	Yes	Yes	Yes
Gao C (73)	2021	HC:28 NMOSD:13 MOGAD:11	HC: 39.64 NMOSD: 41.77 MOGAD:41.09	HC: 68% NMOSD:85% MOGAD: 82%	Yes	Yes	Yes	Yes
Zheng F (74)	2022	HC:280 NMOSD:236	HC: 38.90 NMOSD: 41.09	HC:57% NMOSD:88%	Yes			
Chen X (75)	2021	HC:56 NMOSD:42			Yes			
Cacciaguerra L (76)	2021	HC:101 NMOSD:28	HC: 37.60 NMOSD: 43.60	HC:44% NMOSD:79%	Yes			
Andica C (77)	2022	HC:19 NMOSD:18	HC: 51.47 NMOSD: 52.67	HC:68% NMOSD:89%	Yes			
Rechtman A (78)	2022	HC:22 MOGAD:22	HC: 38.23 MOGAD: 33.10	HC:77% MOGAD:82%		Yes		
Cacciaguerra L (79)	2022	HC:101 NMOSD:72	HC: 42.60 NMOSD: 43.90	HC:71% NMOSD:83%	Yes			
Zhang Y (80)	2022	HC:20 NMOSD:33	HC: 45.20 NMOSD: 44.58	HC:100% NMOSD:94%	Yes			
Cacciaguerra L (81)	2022	HC:27 NMOSD:30	HC: 41.20 NMOSD: 44.10	HC:70% NMOSD:78%	Yes			
Cortese R (82)	2023	HC:34 NMOSD:30 MOGAD:30	HC: 34.7 NMOSD: 40.60 MOGAD:36.90	HC: 71% NMOSD:80% MOGAD: 67%	Yes	Yes	Yes	
Wei R (83)	2023	HC:269 NMOSD:199	HC: 38.50 NMOSD: 41.00	HC:57% NMOSD:88%	Yes			
Lotan I (84)	2023	HC:37 NMOSD:47 MOGAD:24	HC: 40.10 NMOSD: 50.40 MOGAD:39.20	HC: 84% NMOSD:94% MOGAD: 71%	Yes	Yes	Yes	
Xie Y (85)	2023	HC:34 NMOSD:35	HC: 35.79 NMOSD: 42.34	HC:67% NMOSD:80%	Yes			
Bartels F (86)	2023	HC:90 MOGAD:16	HC: No Information MOGAD: 4.51	HC: No Information MOGAD:50%		Yes		
Zakani M (87)	2023	HC:11 NMOSD:9	No Information	No Information	Yes			
Sun J (88)	2023	HC:48 NMOSD:99 MOGAD:28	HC: 36.00 NMOSD: 44.00 MOGAD:36.00	HC: 6% NMOSD:89% MOGAD: 75%	Yes	Yes	Yes	
Masuda H (89)	2023	HC:29 NMOSD:29	HC: 61.00 NMOSD: 59.00	HC:76% NMOSD:76%	Yes			
Ma H (90)	2024	HC:22 NMOSD:18	HC: 44.36 NMOSD: 42.11	HC:86% NMOSD:76%	Yes			
Wang Y (91)	2024	HC:45 NMOSD:30	HC: 41.84 NMOSD: 37.70	HC:73% NMOSD:90%	Yes			
Cortese R (36)	2024	HC:144 NMOSD:135 MOGAD:135	HC: 37.20 NMOSD: 51.10 MOGAD:40.90	HC: 60% NMOSD:82% MOGAD: 61%	Yes	Yes	Yes	
Schneider R (92)	2024	HC:49 MOGAD:23	HC: 31.40 MOGAD: 33.30	HC:53% MOGAD:73.90%		Yes		
Tsai CC (93)	2024	HC:21 NMOSD:17	HC: 42.00 NMOSD: 44.00	HC:71% NMOSD:65%	Yes			

HC, Healthy Control; NMOSD, Neuromyelitis optica spectrum disorder; DTI, diffusion tensor imaging.

### 3.2 Brain volume is significantly lower in NMOSD patients compared to HCs

**Figure 2:**
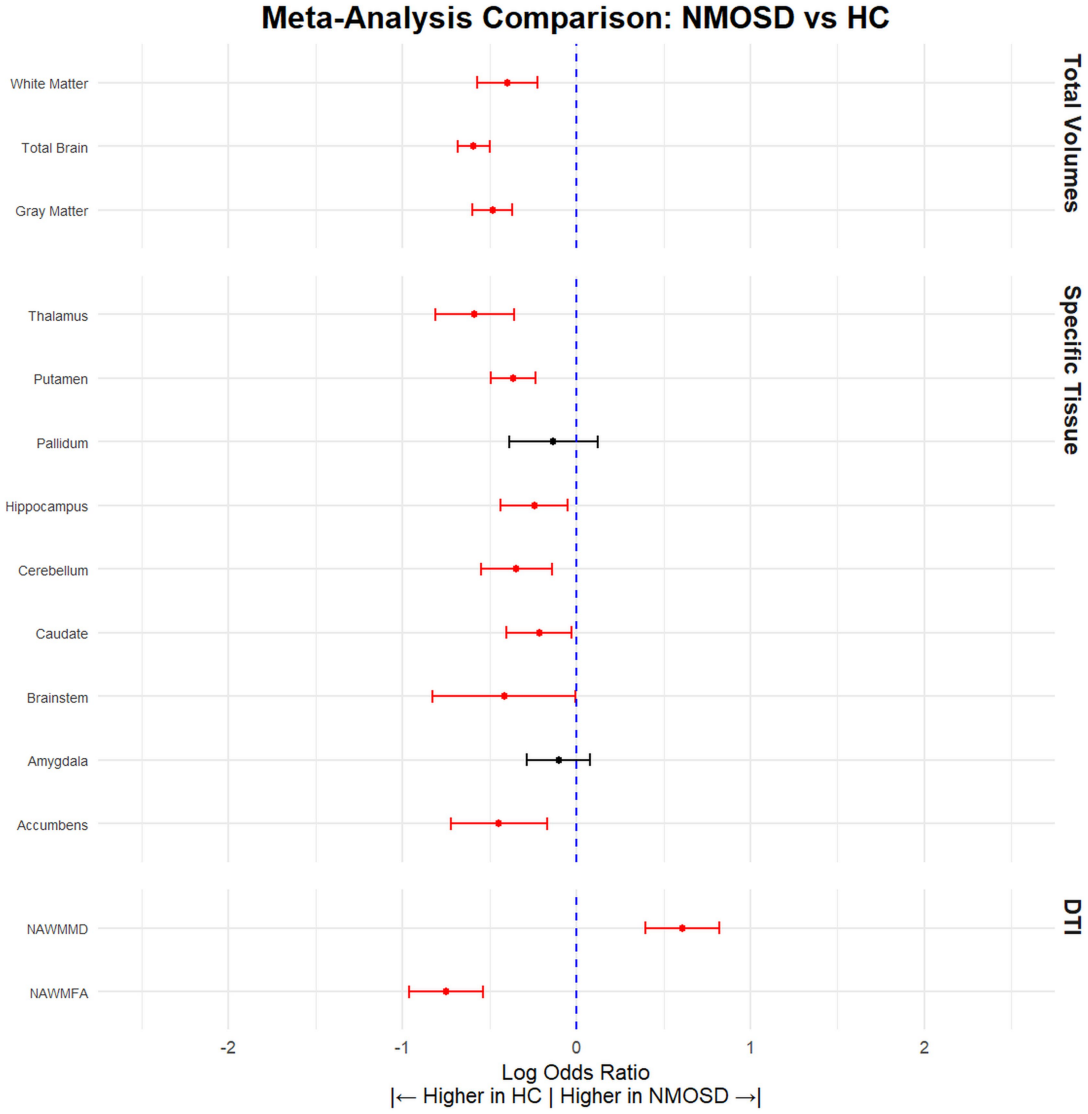
NMOSD patients have significantly lower brain volume compared to HCs. Meta-analysis of volumetric parameters between HCs and NMOSD patients. (Red is significant | Black is not significant) total brain volume (SMD = −0.59, 95% CI [(−0.68) – (−0.50)], *p* < 0.0001, *I*^2^ = 34.31), white matter (SMD = −0.40, 95% CI [(−0.57) – (−0.22)], *p* < 0.0001, *I*^2^ = 75.78), gray matter (SMD = −0.48, 95% CI [(−0.60) – (−0.37)], *p* < 0.0001, *I*^2^ = 50.45), thalamus (SMD = −0.59, 95% CI [(−0.81) – (−0.36)], *p* < 0.0001, *I*^2^ = 77.73), putamen (SMD = −0.36, 95% CI [(−0.49) – (−0.24)], *p* < 0.0001, *I*^2^ = 0.00), pallidum (SMD = −0.13, 95% CI [(−0.39) – 0.12], *p* = 0.30, *I*^2^ = 71.40), hippocampus (SMD = −0.24, 95% CI [(−0.44) – (−0.05)], *p* = 0.01, *I*^2^ = 66.71), cerebellum (SMD = −0.35, 95% CI [(−0.55) – (−0.14)], *p* = 0.0001, *I*^2^ = 27.27), caudate (SMD = −0.22, 95% CI [(−0.40) – (−0.03)], *p* = 0.02, *I*^2^ = 54.06), brainstem (SMD = −0.42, 95% CI [(−0.83) – (−0.01)], *p* = 0.05, *I*^2^ = 70.14), amygdala (SMD = −0.10, 95% CI [(−0.29) – 0.08], *p* = 0.27, *I*^2^ = 28.01), accumbens (SMD = −0.45, 95% CI [(−0.72) – (−0.17)], *p* = 0.002, *I*^2^ = 62.57), normal appearing FA (SMD = −0.59, 95% CI [(−0.96) – (−0.54)], *p* < 0.0001, *I*^2^ = 34.31), and normal appearing MD (SMD = 0.59, 95% CI [0.40–0.82], *p* < 0.0001, *I*^2^ = 34.31).

Our analysis demonstrated that NMOSD patients had significantly lower total brain volume compared to HCs, (SMD = −0.59, 95% CI [(−0.68) – (−0.50)], *p* < 0.0001, *I*^2^ = 34.31). This pattern of reduced volume was consistent across various brain structures. Specifically, the gray matter volume was lower in NMOSD patients (SMD = −0.48, 95% CI [(−0.60) – (−0.37)], *p* < 0.0001, *I*^2^ = 50.45), and white matter volume by (SMD = −0.40, 95% CI [(−0.57) – (−0.22)], *p* < 0.0001, *I*^2^ = 75.78). Furthermore, notable reductions were observed in the volume of the accumbens (SMD = −0.45, 95% CI [(−0.72) – (−0.17)], *p* = 0.002, *I*^2^ = 62.57), brainstem (SMD = −0.42, 95% CI [(−0.83) – (−0.01)], *p* = 0.05, *I*^2^ = 70.14), caudate (SMD = −0.22, 95% CI [(−0.40) – (−0.03)], *p* = 0.02, *I*^2^ = 54.06), cerebellum (SMD = −0.35, 95% CI [(−0.55) – (−0.14)], *p* = 0.0001, *I*^2^ = 27.27), hippocampus (SMD = −0.24, 95% CI [(−0.44) – (−0.05)], *p* = 0.01, *I*^2^ = 66.71), putamen (SMD = −0.36, 95% CI [(−0.49) – (−0.24)], *p* < 0.0001, *I*^2^ = 0.00), and thalamus (SMD = −0.59, 95% CI [(−0.81) – (−0.36)], *p* < 0.0001, *I*^2^ = 77.73) (Figure 2; Table 2).

**Table 2:** Volumetric differences between NMOSD and HCs.

Tissue	Articles (*n*)	HC (*n*)	NMOSD (*n*)	SDM	CILow95	CIHigh95	pValue	*I* ^2^	Leave 1 out (Significane)	Egger pValue
Gray Matter	35	1,475	1,375	−0.48	−0.60	−0.37	*p* < 0.0001	50.45	Stable	0.06
Total Brain	37	1,699	1,695	−0.59	−0.68	−0.50	*p* < 0.0001	34.31	Stable	0.69
White Matter	29	1,273	1,223	−0.40	−0.57	−0.22	*p* < 0.0001	75.78	Stable	0.003
Accumbens	8	366	253	−0.45	−0.72	−0.17	0.002	62.57	Stable	0.008
Amygdala	10	415	293	−0.10	−0.29	0.08	0.27	28.01	Stable	0.37
Brainstem	6	156	193	−0.42	−0.83	−0.01	0.05	70.14	Changed (Not significant)	0.76
Caudate	15	602	500	−0.22	−0.40	−0.03	0.02	54.06	Changed (Not significant)	0.12
Cerebellum	9	253	299	−0.35	−0.55	−0.14	*p* < 0.0001	27.27	Stable	0.13
Hippocampus	16	813	787	−0.24	−0.44	−0.05	0.01	66.71	Stable	0.35
Pallidum	12	526	432	−0.13	−0.39	0.12	0.30	71.40	Changed (Significant)	0.71
Putamen	14	566	464	−0.36	−0.49	−0.24	*p* < 0.0001	0.00	Stable	0.65
Thalamus	22	797	717	−0.59	−0.81	−0.36	*p* < 0.0001	77.73	Stable	0.28
NAWMFA	9	291	267	−0.75	−0.96	−0.54	*p* < 0.0001	26.73	Stable	0.63
NAWMMD	8	242	226	0.61	0.40	0.82	*p* < 0.0001	14.54	Stable	0.57

HC, Healthy Control; NMOSD, Neuromyelitis optica spectrum disorder; CI, confidence interval; SDM, standardized mean difference; NAWMFA, fractional anisotropy normally appearing white matter; NAWMMD, mean diffusivity normally appearing white matter.

### 3.3 Sub-analyses of brain volume reductions in NMOSD versus HCs

#### 3.3.1 Sensitivity analysis excluding overlapping datasets

To assess the potential impact of overlapping datasets from the same research institutions, we conducted a sensitivity analysis excluding studies with a high likelihood of participant duplication. The revised meta-analysis, which included only non-overlapping cohorts, confirmed the primary findings: NMOSD patients continued to show significantly lower brain volumes compared to HCs across nearly all tissue types. Specifically, total brain volume (SMD = −0.62, 95% CI [−0.72 to −0.52], *p* < 0.0001, *I*^2^ = 20.6), gray matter (SMD = −0.44, 95% CI [−0.59 to −0.29], *p* < 0.0001, *I*^2^ = 56.1), and white matter (SMD = −0.40, 95% CI [−0.57 to −0.22], *p* < 0.0001, *I*^2^ = 75.8) remained significantly reduced (Supplementary Table 1).

#### 3.3.2 Assessment of publication bias

To further examine the robustness of our findings, we compared studies that reported significant brain volume reductions in NMOSD versus HCs with those that did not. No differences were observed between the groups in MRI field strength, software used for brain volume quantification, proportion of AQP4-IgG–positive patients, or MRI sequence used for volumetric analysis. We then assessed a subset of studies that initially reported no significant differences in brain volume between NMOSD patients and HCs. Even within this subset, NMOSD patients demonstrated significantly reduced brain volumes, including total brain volume (SMD = −0.52, 95% CI [(−0.66) – (−0.37)], *p* < 0.0001, *I*^2^ = 39.57), gray matter (SMD = −0.42, 95% CI [(−0.63) – (−0.22)], *p* < 0.0001, *I*^2^ = 56.27), and white matter (SMD = −0.26, 95% CI [(−0.44) – (−0.10)], *p* = 0.002, *I*^2^ = 35.90).

#### 3.3.3 Sub-analysis of AQP4-IgG–positive NMOSD cohorts

We next conducted a sub-analysis restricted to studies in which the NMOSD cohort consisted exclusively of AQP4-IgG–positive patients. In this subset, NMOSD-AQP4 + patients continued to exhibit significantly reduced total brain volume (SMD = −0.59, 95% CI [(−0.74) – (−0.43)], *p* < 0.0001, *I*^2^ = 36.82), gray matter volume (SMD = −0.71, 95% CI [(−0.84) – (−0.58)], *p* < 0.0001, *I*^2^ = 14.01), and white matter volume (SMD = −0.14, 95% CI [(−0.26) – (−0.02)], *p* = 0.02, *I*^2^ = 0) compared to HCs. Notably, however, hippocampal volume did not differ between NMOSD-AQP4 + patients and HCs in this analysis (SMD = −0.22, 95% CI [(−0.58) – 0.10], *p* = 0.18, *I*^2^ = 65.70).

#### 3.3.4 Sub-analysis of age- and sex-matched cohorts

Brain volume is strongly influenced by both age and sex. To address these potential confounding factors, we conducted a sub-analysis restricted to studies in which the NMOSD and HC groups were age- and sex-matched. The results were consistent with the primary meta-analysis: NMOSD patients continued to show reduced (SMD = −0.54, 95% CI [(−0.65) – (−0.44)], *p* < 0.0001, *I*^2^ = 25.03), gray matter volume (SMD = −0.48, 95% CI [(−0.59) – (−0.37)], *p* < 0.0001, *I*^2^ = 20.44), and white matter volume (SMD = −0.42, 95% CI [(−0.63) – (−0.21)], *p* = 0.0001, *I*^2^ = 73.82) volumes compared to HCs.

### 3.4 NMOSD patients have altered white matter integrity

DTI analysis revealed altered white matter integrity in NMOSD patients. Specifically, the NAWMFA was significantly lower compared to HCs (SMD = −0.59, 95% CI [(−0.96) – (−0.54)], *p* < 0.0001, *I*^2^ = 34.31). Conversely, the NAWMMD was higher compared to HCs (SMD = 0.59, 95% CI [0.40–0.82], *p* < 0.0001, *I*^2^ = 34.31).

### 3.5 NMOSD patients have lower brain volume compared to MOGAD patients

**Figure 3:**
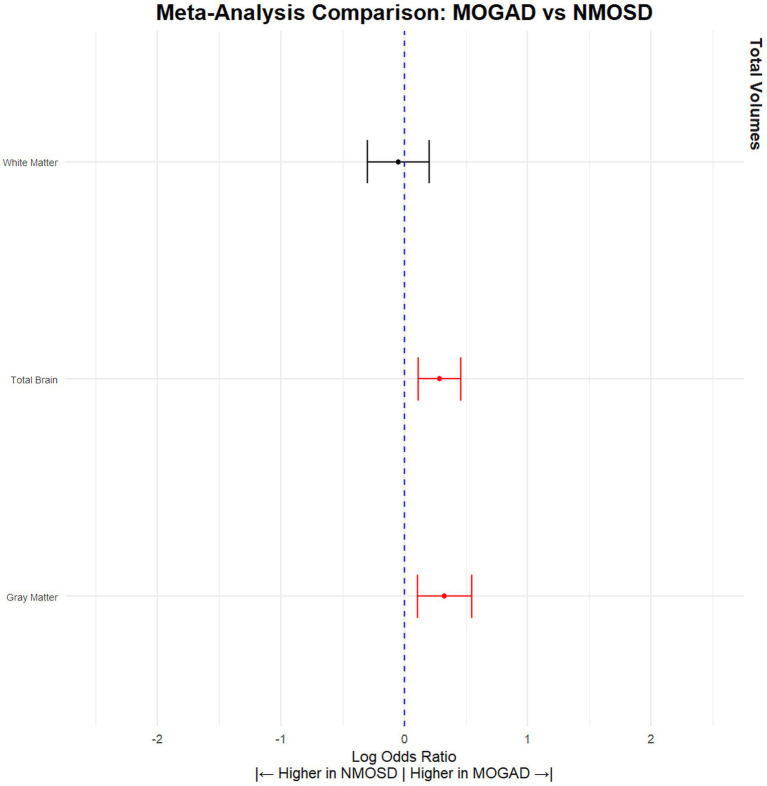
NMOSD patients have significantly lower brain volume compared to MOGAD patients. Meta-analysis of volumetric parameters between NMOSD and MOGAD patients. (Red is significant | Black is not significant) total brain (SMD = −0.28, 95% CI [(−0.11) – (−0.46)], *p* = 0.001, *I*^2^ = 14.68), gray matter (SMD = −0.33, 95% CI [(−0.11) – (−0.55)], *p* = 0.004, *I*^2^ = 43.32), and white matter (SMD = 0.05, 95% CI [(−0.20) – 0.30], *p* = 0.71, *I*^2^ = 50.21).

**Figure 4:**
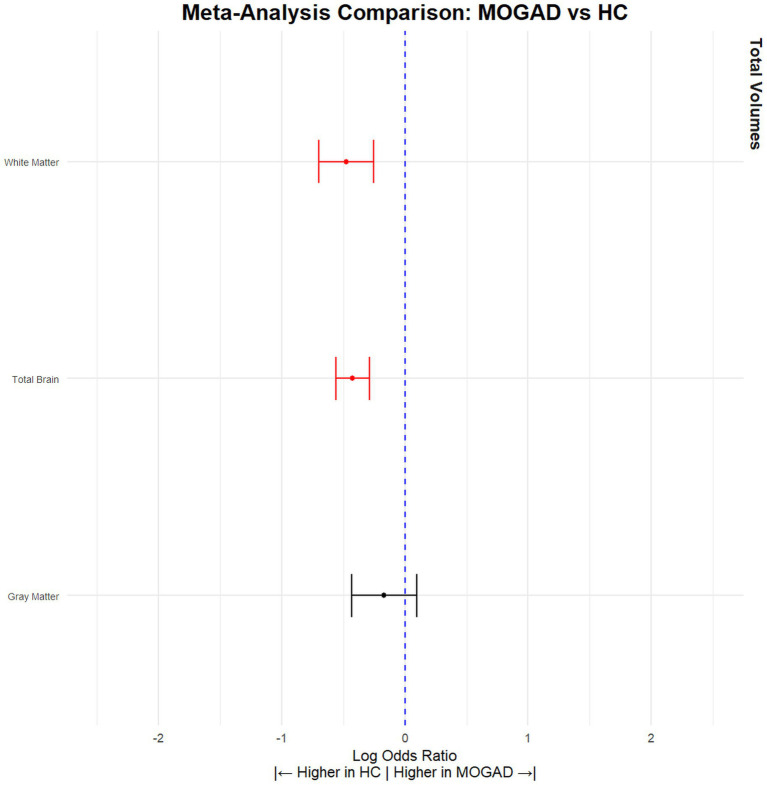
MOGAD patients have significantly lower brain volume compared to HCs. Meta-analysis of volumetric parameters between HCs and MOGAD patients. (Red is significant | Black is not significant) total brain volume (SMD = −0.43, 95% CI [(−0.56) – (−0.29)], *p* < 0.0001, *I*^2^ = 0.00), gray matter volume (SMD = −0.17, 95% CI [(−0.43) – (0.09)], *p* = 0.21, *I*^2^ = 69.36), and white matter volume (SMD = −0.48, 95% CI [(−0.70) – (−0.25)], *p* < 0.0001, *I*^2^ = 52.39).

When comparing NMOSD patients with MOGAD patients, the results indicated that NMOSD patients had significantly lower brain volume (SMD = −0.28, 95% CI [(−0.11) – (−0.46)], *p* = 0.001, *I*^2^ = 14.68) and gray matter volume (SMD = −0.33, 95% CI [(−0.11) – (−0.55)], *p* = 0.004, *I*^2^ = 43.32), but no significant difference was observed in white matter volume (SMD = 0.05, 95% CI [(−0.20) – 0.30], *p* = 0.71, *I*^2^ = 50.21) (Figure 3; Table 3). Conversely, MOGAD patients exhibited lower brain volume (SMD = −0.43, 95% CI [(−0.56) – (−0.29)], *p* < 0.0001, *I*^2^ = 0.00) and white matter volume (SMD = −0.48, 95% CI [(−0.70) – (−0.25)], *p* < 0.0001, *I*^2^ = 52.39) compared to HCs (Figure 4; Table 4).

**Table 3:** Volumetric differences between MOGAD and NMOSD patients.

Tissue	Articles (*n*)	NMOSD (*n*)	MOGAD (*n*)	SDM	CILow95	CIHigh95	pValue	*I* ^2^	Leave 1 out (Significance)	Egger pValue
Total Brain	7	456	307	−0.28	−0.11	−0.46	0.001	14.68	Stable	0.18
Gray Matter	6	443	296	−0.33	−0.11	−0.55	0.004	43.32	Stable	0.10
White Matter	6	396	272	0.05	−0.20	0.30	0.71	50.21	Stable	0.91

NMOSD, Neuromyelitis optica spectrum disorder; MOGAD, myelin oligodendrocyte glycoprotein antibody-associated disease; CI, confidence interval; SDM, standardized mean difference.

**Table 4:** Volumetric differences between MOGAD patients and HCs.

Tissue	Articles (*n*)	HC (*n*)	MOGAD (*n*)	SDM	CILow95	CIHigh95	pValue	*I* ^2^	Leave 1 out (Significance)	Egger pValue
Total Brain	10	362	647	−0.43	−0.56	−0.29	*p* < 0.0001	0	Stable	0.29
Gray Matter	9	351	619	−0.17	−0.43	−0.09	*p* = 0.21	69.36	Stable	0.35
White Matter	8	582	327	−0.48	−0.70	−0.25	*p* < 0.0001	52.39	Stable	0.001

HC, Healthy Control; MOGAD, myelin oligodendrocyte glycoprotein antibody-associated disease; CI, confidence interval; SDM, standardized mean difference.

## 4 Discussion

Our comprehensive meta-analysis, encompassing 61 studies involving substantial numbers of NMOSD, MOGAD patients, and HCs, provided robust quantitative data on brain volumes and white matter integrity across these groups. The results unequivocally demonstrated that NMOSD patients have significantly reduced brain volumes when compared to HCs and MOGAD patients. This reduction spans across total brain volume, gray matter, and specific brain structures including the accumbens, brainstem, caudate, cerebellum, hippocampus, putamen, and thalamus. The consistency of these findings, even in subset analyses that only included studies showing no initial difference between NMOSD patients and HCs, emphasize the pervasive impact of NMOSD on brain structure.

Longer disease duration and frequent relapses can contribute to cumulative neurodegeneration in NMOSD patients, while immunosuppressive therapies may help preserve brain volume by reducing inflammatory activity (25–27). These clinical factors should be considered when interpreting volumetric differences in demyelinating disorders and warrant further investigation. The significant reduction in brain volumes across multiple regions suggests a more extensive neurodegenerative component in NMOSD than previously recognized. This extensive brain volume reduction could potentially contribute to cognitive and neurological deficits observed in NMOSD patients (28). These findings highlights the importance of incorporating brain volume assessments into routine clinical management.

The integration of advanced MRI techniques, such as Neurite Orientation Dispersion and Density Imaging, Diffusion Kurtosis Imaging, Quantitative Susceptibility Mapping, and quantitative myelin imaging, has the potential to significantly enhance our understanding of demyelinating diseases (29–32). These methods enable *in vivo* assessment of microstructural properties, including neurite density, tissue complexity, myelin content, and iron deposition, offering insights beyond the resolution of conventional MRI. As these techniques continue to mature and become more standardized across centers, they are expected to contribute to the development of robust imaging biomarkers that can inform prognosis, stratify patient subgroups, and evaluate therapeutic response with greater precision.

In our meta-analysis, we found that MOGAD patients exhibited significantly lower total brain volumes compared to HCs, despite some individual studies reporting no significant differences. Additionally, when compared to NMOSD patients, individuals with MOGAD demonstrated relatively higher brain volumes. This finding is consistent with the generally milder clinical course observed in MOGAD compared to NMOSD and may reflect differences in underlying disease mechanisms and neurodegenerative burden.

While the meta-analysis revealed consistent patterns of brain volume reduction and altered white matter integrity in NMOSD, we acknowledge that several of the included analyses exhibited moderate to high heterogeneity, with *I*^2^ values exceeding 50% in some cases (e.g., thalamus, pallidum, white matter). This level of heterogeneity is not uncommon in neuroimaging meta-analyses and likely reflects variability in imaging protocols, scanner types, patient demographics, disease stages, and analysis pipelines across studies (33). Despite this variability, the direction and magnitude of effects remained largely consistent. Importantly, certain tissue categories, such as total brain volume (*I*^2^ = 34.31%), and DTI metrics like NAWMFA and NAWMMD (*I*^2^ < 30%)—showed low to moderate heterogeneity, lending greater reliability to these results. These lower-heterogeneity outcomes reinforce the conclusion that brain atrophy and white matter microstructural damage are key features of NMOSD pathology. Nonetheless, the observed heterogeneity in other tissues highlights the need for more standardized imaging methodologies in future studies to further refine these volumetric biomarkers.

MOGAD is a relatively recently recognized clinical entity, and as such, most existing studies are based on small patient cohorts, limiting the generalizability of their findings. In this context, meta-analytical approaches are particularly valuable, as they enable the integration of data across multiple studies, thereby increasing statistical power and improving the reliability of conclusions drawn from larger, aggregated samples. However, the overall scarcity of research, compounded by the typically low number of patients included in individual studies, has contributed to less pronounced findings in MOGAD compared to NMOSD, hindering the robust evaluation of volume differences across various brain tissues. Indeed, several studies have reported no significant differences in brain volume between patients with MOGAD and HCs, although this might largely reflect the small size of cohorts conducted to date. Conversely, NMOSD has been established for a longer period and benefits from a more extensive literature base, which is why the current study primarily focused on NMOSD.

We also compared brain volume in a subcohort of only AQP4 + patients. We observed reduced total, gray, and white matter volumes, with significant atrophy in the thalamus and putamen. In contrast to the full NMOSD cohort, the hippocampus did not show significant differences, which may reflect underlying pathophysiological differences between AQP4-positive and AQP4-negative patients, or may be due to the smaller number of studies included in this comparison.

Leave-one-out analyses demonstrated that the direction of effect sizes was stable across most tissues. In the majority of regions, statistical significance also remained consistent. Only three regions (brainstem, caudate, and pallidum in the NMOSD vs. HC comparison) exhibited changes in significance depending on the excluded study, suggesting some sensitivity to individual datasets. Egger’s test indicated potential publication bias in a subset of regions, particularly in white matter and accumbens, while most regions showed non-significant results, indicating limited evidence of small-study effects overall. The significant Egger’s test for white matter highlights the need for cautious interpretation of findings in this region, especially in studies with smaller sample sizes.

To address the potential concern of dataset redundancy, particularly due to multiple studies originating from the same research institutions, we conducted a sensitivity analysis excluding studies with a high likelihood of overlapping patient cohorts. Despite the reduction in the number of included studies, the main findings remained consistent in both direction and statistical significance, particularly for major volumetric measures such as total brain volume, gray matter, white matter, and several deep gray matter structures. These consistent results reinforce the robustness of our conclusions and strengthen the evidence for a widespread neurodegenerative process in NMOSD. Although it remains challenging to definitively verify patient overlap, especially given the complexity of multi-center collaborations, this additional analysis mitigates the risk of bias from duplicated samples and provides further confidence in the reliability of the primary findings.

Variability in brain volume estimates across software packages such as FreeSurfer, FSL, and SPM is well-documented and complicates cross-study comparisons due to differences in segmentation algorithms and anatomical templates (34). Additional variability arises from differences in MRI scanner type, field strength, and acquisition protocols (35). Meta-analyses that include studies using diverse tools are valuable, as they can uncover consistent disease-related patterns and improve generalizability in NMOSD research. For longitudinal studies within the same patient, however, maintaining consistency in scanner, sequence, and software is critical to reduce technical bias.

Although most studies included in our meta-analysis applied lesion-filling procedures to minimize segmentation errors, the potential influence of ex vacuo atrophy remains a relevant consideration. This phenomenon is well documented in MS, where larger T2 lesion volumes have been shown to correlate with increased brain atrophy, likely reflecting tissue collapse or fluid shift following focal white matter damage (36). In NMOSD and MOGAD this effect is expected to be less pronounced due to the generally lower lesion burden observed in most patients (36). However, even in these disorders, the potential for lesion-driven structural changes should be acknowledged in future volumetric studies to ensure accurate attribution of observed atrophy patterns.

The strength of our study lies in its large sample size and the rigorous methodological approach, including a comprehensive search strategy and stringent inclusion criteria, which enhance the generalizability of our findings. However, our analysis is not without limitations. The heterogeneity in imaging protocols and analysis methods across studies might have introduced variability in the measurements. Additionally, while the inclusion of both whole-brain and region-of-interest studies may increase methodological variability, we chose to include both to avoid potential selection bias and to provide a more comprehensive overview of the literature. Furthermore, the retrospective nature of most included studies limits the ability to infer causation.

In conclusion, our meta-analysis confirms significant neurodegeneration in NMOSD patients compared to HCs and MOGAD patients, with substantial reductions in brain volume and alterations in white matter integrity. These findings suggest that NMOSD may have a more pronounced neurodegenerative impact than previously understood.

## Data Availability

The raw data supporting the conclusions of this article will be made available by the authors, without undue reservation.
