# Supplementary material for: Brain atrophy in NMOSD and MOGAD: a meta-analysis of volumetric and DTI biomarkers
Source: Front Neurol. 2025 Dec 2;16:1703283. doi: 10.3389/fneur.2025.1703283 (PMC12705398; doi:10.3389/fneur.2025.1703283)
Supplement: Supplementary file 1 [file Table_1.docx]

| Tissue | Articles | HC (n) | NMOSD (n) | LogOR | LowerCI_LogOR | UpperCI_LogOR | pValue | I2 |
| --- | --- | --- | --- | --- | --- | --- | --- | --- |
| Total_Brain | 24 | 1199 | 1190 | -0.62 | -0.72 | -0.52 | <0.0001 | 20.56 |
| Gray_Matter | 25 | 1033 | 944 | -0.44 | -0.59 | -0.29 | <0.0001 | 56.14 |
| White_Matter | 29 | 1273 | 1223 | -0.40 | -0.57 | -0.22 | <0.0001 | 75.78 |
| Accumbens | 5 | 232 | 143 | -0.67 | -1.03 | -0.30 | <0.0001 | 62.07 |
| Amygdala | 7 | 281 | 183 | -0.07 | -0.28 | 0.13 | 0.48 | 13.25 |
| Brainstem | 6 | 156 | 193 | -0.42 | -0.83 | -0.01 | 0.05 | 70.14 |
| Caudate | 15 | 602 | 500 | -0.22 | -0.40 | -0.03 | 0.02 | 54.06 |
| Cerebellum | 9 | 253 | 299 | -0.35 | -0.55 | -0.14 | <0.0001 | 27.27 |
| Hippocampus | 16 | 813 | 787 | -0.24 | -0.44 | -0.05 | 0.01 | 66.71 |
| Pallidum | 12 | 526 | 432 | -0.13 | -0.39 | 0.12 | 0.30 | 71.40 |
| Putamen | 14 | 566 | 464 | -0.36 | -0.49 | -0.24 | <0.0001 | 0.00 |
| Thalamus | 22 | 797 | 717 | -0.59 | -0.81 | -0.36 | <0.0001 | 77.73 |
| NAWMFA | 9 | 291 | 267 | -0.75 | -0.96 | -0.54 | <0.0001 | 26.73 |
| NAWMMD | 8 | 242 | 226 | 0.61 | 0.40 | 0.82 | <0.0001 | 14.54 |

**Supplementary Table 1:** Meta-Analysis results without suspected duplicated samples
